# Risk Factors of Antibiotic Misuse for Upper Respiratory Tract Infections in Children: Results from a Cross-Sectional Knowledge-Attitude-Practice Study in Greece

**DOI:** 10.5402/2012/685302

**Published:** 2012-11-01

**Authors:** Sotiria G. Panagakou, Vassiliki Papaevangelou, Adamos Chadjipanayis, George A. Syrogiannopoulos, Maria Theodoridou, Christos S. Hadjichristodoulou

**Affiliations:** ^1^Department of Hygiene and Epidemiology, Faculty of Medicine, University of Thessaly, Thessaly, 41222 Larisa, Greece; ^2^Second Department of Paediatrics, Aglaia Kyriakou Children's Hospital, University of Athens, 11527 Athens, Greece; ^3^Paediatric Department, Larnaca General Hospital, 6043 Larnaca, Cyprus; ^4^First Department of Pediatrics, Faculty of Medicine, University of Thessaly, 41110 Larisa, Greece; ^5^Department of Paediatrics, Agia Sofia Children's Hospital, University of Athens, 11527 Athens, Greece

## Abstract

*Background*. Upper respiratory tract infections (URTIs) are common in children. The cause of URTIs is usually viral, but parents' attitudes often contribute to inappropriate prescription of antibiotics, promoting antibiotic resistance. The objective of this study was to identify possible risk factors associated with antibiotic misuse in Greece, a country with high levels of antibiotic use and antibiotic resistance. *Methods*. A knowledge-attitude-practice (KAP) questionnaire was developed and distributed to Greek parents caring for children who were 5-6 years old, between January and July of the same school year. *Results*. The sample of the study contained 5312 parents from all geographic areas of Greece. The risk factors of being a father, having low education, having immigrant status, being a single parent, having low income, having <2 or >3 children, living in the islands, and being without experience in recurrent URTIs were significantly associated to inadequate knowledge, inappropriate attitudes, and wrong practices. *Conclusions*. This study has identified the main groups of parents that should be targeted in future intervention programs.

## 1. Introduction 

Upper respiratory tract infections (URTIs) in children are mainly due to viral infections [1, 2]. Thus, the benefit from antimicrobial drugs is minimal. However, there is strong evidence that antibiotics are frequently administered to children suffering from URTIs [3, 4]. It appears that both pediatricians and parents are responsible for this antibiotic misuse [5–7] which is contributing to the development of resistant strains of bacterial pathogens [8–10] and placing a burden on the economy of the health care system. 

Amongst European countries, Greece presents the highest antibiotic consumption and antibiotic resistance [11]. In order to reveal the contributing factors of this phenomenon, a knowledge, attitude, and practice (KAP) study of Greek parents towards antibiotic use in children suffering from URTIs was performed. The objective of the current study was to identify possible risk factors associated with antibiotic misuse. This would enable the development of intervention strategies aiming to parental education. 

## 2. Materials and Methods

### 2.1. Study Design and Settings

The study sample comprised of parents from all geographic areas of Greece. A school-based stratified geographic clustering sampling was used to select a representative sample of students. Seven thousand seven hundred and four parents (7704) of children aged between 5 and 6 years old participated in the survey. Approval was given by the Ministry of Education in order to contact the parents through the school system and to distribute anonymous questionnaires. Due to the anonymity of the questionnaire (available online at doi:10.5402/2012/685302 as supplementary material), written informed consent from parents was waived. The survey was conducted between January and July 2007. All methodological aspects of the study, including the sampling, design-development-completeness-reliability of the questionnaire, and any limitations which emerged, have been presented in previous papers [12, 13]. 

In the current study an effort was made to identify risk factors associated with injudicious antibiotic use. To accomplish this aim, a subsample of questions which suggested antibiotic misuse from each section was selected. Therefore, to increase the sensitivity of our survey, three groups of questions were formed: questions Q17, Q18, Q19 and Q22 (knowledge section), Q30, Q31, Q32, Q34, (attitude section), and questions Q42, Q45, Q46 (practice section). The questionnaire has been added as an annexure in the manuscript. Parents giving an incorrect answer to any of the questions included in a group were considered as being erroneous in their response for the entire group of questions. Additionally, questions Q16 (knowledge section), Q27, and Q28 (attitude section) were examined separately since they contained a number of subquestions. Whether specific parental demographic characteristics are associated with incorrect answering to the selected questions was examined. In order to assess the quality of the results obtained with the method described above, we also combined the questions in scales in order to give overall attitude, knowledge, and practice scores. The scores were afterwards associated with parental demographic characteristics.

### 2.2. Data Analysis

The data was entered into a database using the Epi Info program. Statistical Package for Social Sciences (SPSS) version 15.0 was used to analyze data from the questionnaire. Chi-square test and Kruskal-Wallis test were used to assess statistical significant correlations between the variables. Ninety-five percent confidence intervals were calculated for relative risks. The level of significance was 0.05. 

Backward logistic regression was used to identify independent factors associated to injudicious antibiotic use. During backward logistic regression in the 5-point Likert scales, the answers “strongly agree” and “agree” were considered as a “positive” answer, while the answers “disagree” and “strongly disagree” were considered as a “negative” answer. The answer “uncertain” was not taken into consideration. The answers “always,” “most of the times,” and “often” were considered as “frequently”, while the answers “sometimes” and “never” were considered as “rarely.” 

## 3. Results

Five thousand three hundred and twelve questionnaires were collected out of 7704 that were initially disseminated, representing a response rate of 68.95%. Tables 1, 2, and 3 show the percentile number of the parents who gave wrong answers to the selected questions in the sections of knowledge, attitude, and practices, respectively, according to the univariate analysis. 

Table 4 describes the results of the backward logistic regression analysis performed. The risk factors of father, low education, and immigrant status were detected as significantly associated for all the sections (knowledge-attitudes-practices). Additional risk factors were revealed in the knowledge section when the latter was examined separately (parents living in the islands, having low income, <2 or >3 children, and no experience in recurrent URTIs) while questions from the attitude section were significantly related additionally to the demographic characteristics of being a single parent and having no experience in recurrent URTIs. The same results were derived even when we combined the questions in scales in order to give overall attitude, knowledge, and practice scores (data not shown).

## 4. Discussion

Even though it has been widely recognized that URTIs are the most often of viral etiology [14] and clinical practice guidelines for their management are well established, still antibiotics are prescribed for children with URTIs [15–18]. Greece is one of the countries with the highest antibiotic consumption in Europe [11]. Additionally, a recent European Commission report indicated that Greece also has the highest over the counter antibiotic sales amongst 27 EU countries [19]. In Greece, parents have free access to all types of ordinary antibiotics despite a specific legislation forbidding antibiotic use without a prescription. In a recent survey that took place in the capital of Greece, Athens, volunteers presented to pharmacies asking for ciprofloxacin and co-amoxiclav to document if it is possible to obtain antibiotics without a prescription [20]. Co-amoxiclav was given in 100% of cases while ciprofloxacin was given in 53%, pointing by this way out the extent of just one aspect of antibiotic misuse. Our study [13] has shown that over—the—counter use was very low but it is unclear how this reflects real-life practice. 

Since there is evidence indicating that antibiotic overuse drives bacterial resistance, [8–10] the identification of factors influencing antimicrobial prescription in pediatric practice may have a considerable public health impact. This is the first population-based KAP study of Greek parents and the current paper aims to describe parental risk factors associated with antibiotic overuse in Greece.

This study was able to portray the demographic profile of parents prone to antibiotic misuse. The main parental demographic characteristics associated with antibiotic misuse include being a father, having low educational level, and being an immigrant. 

There have been several studies indicating a positive relationship between parental educational level and their expected knowledge about antibiotic treatment [21–23]. It is probable that low education is related to inadequate information about judicious antibiotic use which can lead to improper practices [22, 24]. Indeed, as shown in Tables 2, 3, and 4, parental educational level is inversely related to the percentage of parents answering incorrectly. Limited access to Internet, other media, or literature among poorly educated parents maybe related to reduced source availability concerning antibiotic consumption. On the contrary, parents with higher educational status seem to better acknowledge the risks of antibiotic misuse, making pediatricians more skeptical to offer antibiotic treatment [25]. Furthermore, being an immigrant was associated with antibiotic misuse. In Greece, minorities represent more than 10% of population and a similar percentage was assessed among the participants in the study (10%). The vast majority of immigrants in Greece are of low socioeconomic status, possibly explaining their pressure for antibiotic therapy as aforementioned. In addition, being away from homeland often causes uncertainty and high worry of a child's illness, leading to antibiotic demand. Moreover, as many immigrants are not fluent in Greek, it is possible that miscommunication among parents and pediatricians in reference to medical history and proper treatment may also contribute to increased antibiotic consumption [26]. Finally, cultural beliefs vary among minorities. Mangione-Smith et al. in Los Angeles found that non-Hispanic white parents were less likely to expect antibiotics than Latino, Asian, and African American parents [26]. Other studies have reported the immigrant status as being a positive predictor of receiving an antibiotic prescription too [24, 27, 28].

Interestingly, a strong risk factor associated with all the three sections of the questionnaire was being a father. One could postulate that Greek fathers do not participate as much as mothers in upbringing their children and therefore they may be less inclined to research information concerning medical matters. Fathers' participation in the daily care of their children has significantly changed over the past decades due to the increase in the number of women entering the labor force, the increase of one-parent families, the decrease of married couples, the decrease in the number of children in nuclear families, and the spread of the child-centered ideology [29]. However, in a recent study among Greek fathers, there was evidence that fathers from rural areas with low academic achievements and occupational status are less likely to contribute to child care practices than fathers living in urban areas with high educational and occupational status [30]. To our knowledge, such correlation has not been mentioned in other studies and could be due to cultural issues. 

Additional risk factors were detected in the knowledge and attitude section. Single parents reported improper attitudes concerning antibiotic use. As it has also been acknowledged [25, 31] that it is more difficult to take care of a sick child in single-parent families, especially if other relatives are not available. Thus, one could speculate that single parents expect antibiotic treatment assuming that it will shorten disease duration and therefore allowing them to return to their workplace earlier. As expected, limited parental experience to recurrent URTIs was also found to be a determinant of antibiotic misuse. Probably as parents get exposed to more incidents of URTIs, their chance to receive education concerning judicious antibiotic use increases.

Moreover, island inhabitants tended to give incorrect answers. Greek geographical area contains several islands, most of which suffer from a weak healthcare network, which is most likely unable to properly educate the inhabitants about antibiotic misuse. Moreover, in many islands, access to hospitals is difficult, which is increasing pediatricians' anxiety and their prescribing antibiotics for fear of possible secondary complications after a URTI.

Low income was also described as a risk factor driving to antibiotic misuse. Low educational status can often be related to low household income as depicted in other studies [31, 32]. In addition, jobs with lower reimbursement usually have less working hours flexibility, discouraging parents to spend time with their sick child at home and therefore leading them to administer antibiotic therapy in hope that the child will recover quicker and let them return to work [32]. 

Finally, having either one or more than three children was associated with poor antibiotic knowledge. Kuzujanakis et al. also associated adequate antibiotic knowledge with having >1 child [28]. It was suggested that parents gain experience concerning antibiotic use when dealing with more than one child. However, it is difficult to explain why parents with more than 3 children presented poor antibiotic knowledge scores. One could postulate, that families with >3 children tend to be of low socioeconomic status although no such evidence has been reported elsewhere.

However, amongst various studies that have been held in other countries, additional risk factors had been identified. Parental age has been reported as a factor affecting antibiotics expectation [25, 28], as well as the type of insurance [27]. Even though in our study these factors were included in the demographic characteristics and were elaborated through analysis, significant statistical association after the logistic regression analysis was not observed. Finally, in our study, parents reporting a typical relationship with their pediatrician were not more likely to provide incorrect answers but often failed to identify common antibiotic names.

An effort was made to identify the limitations of this study. The survey was conducted from January to June. Winter and spring seasons are generally associated with an increased likelihood of URTIs and therefore antibiotic prescriptions, therefore parents may have reported an overestimating practice of antibiotic overuse. Another limitation concerns the assertion on parents' self-report about their knowledge, attitude, and practices towards antibiotic use. As parents were informed that this questionnaire was used for a study, they might have been reluctant to report practices that could be considered inappropriate. An additional limitation of the study refers to the language and medical terms used when designing the questionnaire. Although an effort was made to use simple words, parents with lowest education and immigrants might have been unable to comprehend the questionnaire.

This study has identified the main groups of parents to which intervention programs should aim. Thus, in Greece, future campaigns should be targeting mostly parents of low educational status, immigrants and fathers. However the role of pediatricians themselves in reducing antibiotic misuse should not be underestimated. In fact, our study [13] showed that parents play a much lesser role than paediatricians on antibiotic overuse, indicating that determining what doctors practice for URTI treatment is probably the key to tackle this major issue, as also described in intervention programs [33]. Therefore, such campaigns should focus on educating parents and paediatricians as well about the role of antibiotics, the cost effectiveness of their administration, and the significant problem of antibiotic resistance in the community. 

## Supplementary Material

Questionnaire that was disseminated to parents nationwide.

## Figures and Tables

**Table 1:** Parents (%) who did not answer correctly in the selected questions of the knowledge section.

	Q15	*P* value	Q17, Q18, Q19, Q22	*P* value
Athens	**8.4**	0.004*	**1.7**	0.004*
Northern Greece	**9.7**		**1.9**	
Central Greece	**9.2**		**3.1**	
Islands versus	**13.9**		**3.8**	
Peloponnese	**9.1**		**1.3**	

Mothers	**6.9**	**<**0.001*	**1.6**	**<**0.001*
Fathers	**19**		**4.1**	

31–45 years	**9.1**	**<**0.001*	**1.9**	0.006*
<31 or >45 years	**13.5**		**3.5**	

Insurance				
Public	**9.4**	0.001*	2.1	0.29
Private	**16.3**		1.7	
Public and private	**8.3**		2.6	
None	**31.3**		12.5	

Education of father				
Primary school	**17**	**<**0.001*	**4.3**	**<**0.001*
Secondary school	**15.7**		**2.9**	
High school	**9.3**		**1.9**	
College	**5.3**		**1.5**	
University	**4.1**		**0.5**	

Education of mother				
Primary school	**24.4**	**<**0.001*	**7.9**	**<**0.001*
Secondary school	**22.6**		**4.6**	
High school	**8.8**		**2.1**	
College	**4.1**		**0.8**	
University	**4.3**		**0.5**	

Income^∧^				
High	**5.6**	**<**0.001*	**1.3**	**<**0.001*
Moderate	**8.5**		**2**	
Low	**18.8**		**4.3**	

Immigrants	**35.9**	**<**0.001*	**10.3**	**<**0.001*
Nonimmigrants	**6.7**		**1.3**	

Residence				
Big Towns	**8.8**	0.002*	2.2	0.773
Small Towns	**10.3**		2	
Villages	**13.1**		2.4	

Number of children				
2-3	**8.7**	**<**0.001*	2.2	0.513
<2 *ή* >3	**13.4**		2.2	

Couples	9.4	0.217	2.1	0.056
Singles	11		3.7	

Experience of recurrent URIs				
Yes	**4.2**	**<**0.001*	2.5	0.256
No	**10.6**		2.1	

Pediatrician-parent relation				
Friends or relatives	**8.3**	0.009*	2	0.318
Typical	**10.6**		2.2	

Access to healthcare system				
Good	**11.5**	**<**0.001*	**3**	0.003*
Moderate	**8.4**		**1.6**	
Poor	**5.7**		**1.2**	

Bold letters and *: variables with elevated level of significance after univariate analysis.

Income^∧^: self-assessment as perceived by the parents at the time of the survey.

**Table 2:** Parents (%) who did not answer correctly in the selected questions of the attitude section.

	Q27	*P* value	Q28	*P* value	Q30, Q31, Q32, Q34	*P* value
Athens	**31.7**	0.000*	6.7	0.154	0.2	0.106
Northern Greece	**36.6**		6		0.2	
Central Greece	**44.2**		7.5		0.4	
Islands versus	**38.8**		7.9		0.6	
Peloponnese	**35.8**		5.1		0	

Mothers	**35.7**	0.005*	**5.4**	**<**0.001*	0.2	0.414
Fathers	**40.2**		**10.5**		0.3	

31–45 years	**35.7**	**<**0.001*	**6.2**	0.015*	0.2	0.293
<31 or >45 years	**41.9**		**8.3**		0.4	

Insurance						
Public	**37.2**	0.049*	6.4	0.779	0.3	0.467
Private	**32.5**		5.7		0	
Public and private	**34.3**		6.5		0	
None	**62.5**		12.5		0	

Education of father						
Primary school	**45**	**<**0.001*	**12**	**<**0.001*	0.3	0.572
Secondary school	**41**		**9.1**		0.3	
High school	**36.9**		**5.8**		0.2	
College	**33.6**		**5**		0.1	
University	**31.8**		**3.9**		0	

Education of mother						
Primary school	**45.2**	**<**0.001*	**14.4**	**<**0.001*	0.3	0.398
Secondary school	**44.4**		**10.1**		0.3	
High school	**36.8**		**5.9**		0.4	
College	**34.8**		**4.7**		0.1	
University	**31.9**		**4.7**		0.1	

Income^∧^						
High	**33.5**	**<**0.001*	**5**	**<**0.001*	0.1	0.626
Moderate	**35.8**		**6.1**		0.2	
Low	**44.7**		**11.2**		0.4	

Immigrants	**55.8**	**<**0.001*	**18.3**	**<**0.001*	**1.2**	0.000*
Nonimmigrants	**34.7**		**5.2**		**0.1**	

Residence						
Big Towns	36.1	0.271	6.4	0.625	0.2	0.946
Small Towns	37.6		6.5		0.2	
Villages	39.1		7.4		0.3	

Number of children						
2-3	36.8	0.413	**6**	0.009*	0.2	0.299
<2 *ή* >3	36.4		**8**		0.3	

Couples	**36.4**	0.043*	**6.3**	0.033*	0.2	0.494
Singles	**41.5**		**9.3**		0	

Experience of recurrent URIs						
Yes	**40.9**	0.005*	6.3	0.478	0.1	0.53
No	**36**		6.5		0.2	

Pediatrician-parent relation						
Friends or relatives	37.1	0.263	6	0.057	0.3	0.449
Typical	38.2		7.4		0.2	

Access to healthcare system						
Good	**39**	0.016*	**7.3**	0.038*	0.2	0.895
Moderate	**35.2**		**5.4**		0.3	
Poor	**34.5**		**7**		0.2	

Bold letters and *: variables with elevated level of significance after univariate analysis.

Income^∧^: self-assessment as perceived by the parents at the time of the survey.

**Table 3:** Parents (%) who did not answer correctly in the selected questions of the practice section.

	Q42, Q45, Q46	*P* value
Athens	**25.5**	0.005*
Northern Greece	**22.5**	
Central Greece	**27.5**	
Islands versus	**24.5**	
Peloponnese	**20.2**	

Mothers	**22.5**	**<**0.001*
Fathers	**28.8**	

31–45 years	**23.1**	0.002*
<31 or >45 years	**28**	

Insurance		
Public	23.5	0.372
Private	22	
Public and private	25.4	
None	37.5	

Education of father		
Primary school	**30.5**	**<**0.001*
Secondary school	**26.7**	
High school	**23.9**	
College	**22.7**	
University	**19.6**	

Education of mother		
Primary school	**36.9**	**<**0.001*
Secondary school	**29**	
High school	**25.5**	
College	**19.3**	
University	**19.7**	

Income^∧^		
High	**22.6**	0.001*
Moderate	**23.2**	
Low	**30.5**	

Immigrants	**43.7**	**<**0.001*
Nonimmigrants	**21.8**	

Residence		
Big Towns	23.7	0.661
Small Towns	24.8	
Villages	23.2	

Number of children		
2-3	23.7	0.25
<2 *ή* >3	24.7	

Couples	23.8	0.355
Singles	24.9	

Experience of recurrent URIs		
Yes	22.2	0.132
No	24.1	

Pediatrician-parent relation		
Friends or relatives	26.1	0.052
Typical	23.7	

Access to healthcare system		
Good	**24.8**	0.028*
Moderate	**23.4**	
Poor	**19.5**	

Bold letters and *: variables with elevated level of significance after univariate analysis.

Income^∧^: self-assessment as perceived by the parents at the time of the survey.

**Table 4:** Backwards logistic regression analysis of the selected questions.

	Q15		Q17, Q18, Q19, Q22		Q27		Q28		Q30, Q31, Q32, Q34		Q42, Q45, Q46	
	OR	*P* value	OR	*P* value	OR	*P* value	OR	*P* value	OR	*P* value	OR	*P* value
Islands versus	1.409	0.041*	0.546	**0.016**	0.958	0.644					1.062	0.562
other Greek regions												

Fathers versus	0.347	**<**0.001*	2.232	**<**0.001*	1.119	0.139	1.798	**<**0.001*			1.257	0.005*
mothers												

<31 or >45 years versus	1.278	0.109	0.994	0.469	0.864	0.071	0.834	0.224			0.848	0.063
31–45 years												

Uninsured versus	3.119	0.089			0.724	0.573						
insured												

School graduates mothers versus	1.745	**<**0.001*	0.362	**<**0.001*	0.916	0.194	0.817	0.14			0.753	**<**0.001*
upper education of mothers												

School graduates fathers versus	1.215	0.178	1.644	0.032*	0.876	0.049*	0.861	0.269			1.037	0.631
upper education of fathers												

Income^∧^ high or moderate versus	0.516	**<**0.001*	1.441	0.118	1.074	0.409	1.131	0.414			1.07	0.474
low income												

Immigrants	5.925	**<**0.001*	0.163	**<**0.001*	0.461	**<**0.001*	0.298	**<**0.001*	0.089	**<**0.001*	0.407	**<**0.001*
Nonimmigrants												

Urban versus	0.82	0.116										
rural												

<2 *ή* >3 children versus	0.641	0.001*					1.232	0.119				
2-3 children												

Singles versus					1.333	0.025*	1.709	0.015*				
couples												

No experience of recurrent URIs versus	0.374	**<**0.001*			0.777	0.002*						
experience of recurrent URIs												

Typical pediatrician-parent relation versus	0.943	0.669										
intimate pediatrician-parent relation												

Unsatisfactory access to healthcare system versus	1.087	0.639	0.848	0.59	0.918	0.297	1.19	0.259			0.969	0.733
satisfactory access to healthcare system												

Bold letters and *: variables with elevated level of significance after backward logistic regression analysis.

Income^∧^: self-assessment as perceived by the parents at the time of the survey.
