# Supplementary material for: Risk Factors of Antibiotic Misuse for Upper Respiratory Tract Infections in Children: Results from a Cross-Sectional Knowledge-Attitude-Practice Study in Greece
Source: ISRN Pediatr. 2012 Nov 1;2012:685302. doi: 10.5402/2012/685302 (PMC3503327; doi:10.5402/2012/685302)
Supplement: Supplementary file 1 — Questionnaire that was disseminated to parents nationwide. [file 685302.f1.doc]

**QUESTIONNAIRE**

**Demographic characteristics**

1. Sex: Male

Female

1. Age: ____ years old
2. Assurance: Public

Private

Both

None

1. Type of public insurance:____________

1. Parents’ educational status:

|  | Mother | Father |
| --- | --- | --- |
| Primary School |  |  |
| Secondary School |  |  |
| High School |  |  |
| College |  |  |
| University – Postgraduate studies |  |  |

1. Would you describe your

| Very high | High | Μoderate | Low | Very Low |
| --- | --- | --- | --- | --- |
|  |  |  |  |  |

family income level as:

1. Are you immigrant in Greece? No

Yes

1. In case of immigrance, your native country is: ____________
2. Are you habitants of: Big town

Small town

Village

1. Number of children: ____
2. Are you a single parent? No

Yes

1. Does your child suffer often from chronic diseases involving the respiratory system (such as asthma)?

Yes

No

1. Does your pediatrician happen to be: Family relative

Friend

Just professional relation

1. Would you consider your

| Very good | Good | Μoderate | Bad | Very bad |
| --- | --- | --- | --- | --- |
|  |  |  |  |  |

access to health services as:

**Section A**

1. Sources of information you have about judicious antibiotic use:

Physician

Television

Radio

Newspaper

Friend

Family relative

Job

Other: ___________

1. Which one of the following drugs are antibiotics?

Augmentin

Depon

Aerolin

Ceclor

Ponstan

Amoxill

Mucosolvan

Erythrocin

|  | Strongly agree | Agree | Uncertain | Disagree | Strongly disagree |
| --- | --- | --- | --- | --- | --- |
| 1. Antibiotic should be given to all children who develop a fever. |  |  |  |  |  |
| 1. As most of the Upper Respiratory Tract Infections (like cold, flue, sore throat, ear infection) are of viral origin, antibiotics should not be given because they are self - limited. |  |  |  |  |  |
| 1. Children with flu like symptoms get better faster when antibiotics are given |  |  |  |  |  |
| 1. Scientists can produce new antibiotics that can kill the resistant bacteria. |  |  |  |  |  |
| 1. Antibiotics do not have any side - effects. |  |  |  |  |  |
| 1. Inappropriate use of antibiotics reduces their efficacy and drives bacterial resistance. |  |  |  |  |  |
| 1. Antibiotic use can prevent complications from Upper Respiratory Tract Infections. |  |  |  |  |  |

**Section B**

1. How many days would you let pass in order to visit a pediatrician, if your child presents some symptoms (ie. Nose drainage, sore throat, vomit, cough, fever)? ________days
2. What kind of therapy - ies would you expect from your pediatrician to suggest for your child when it suffers from an Upper Respiratory Infection?

Antibiotics

Analgesics – antipyretics

Antitussives

Antistamines

Normal Serum

Inhalers

Other: ________

1. Which one – s of the following symptoms would make you visit a pediatrician for your child?

Cough

Fever

Nose drainage

Ear pain

Sore throat

Hoarseness

Change of behavior

Other: __________

| 1. How often would you like your pediatrician to prescribe antibiotics for your child when it suffers from: | Always (95-100%) | Most of the times (70-95%) | Often  (30-70%) | Sometimes (5-30%) | Never  (0-5%) |
| --- | --- | --- | --- | --- | --- |
| Cold |  |  |  |  |  |
| Nose drainage |  |  |  |  |  |
| Sore throat |  |  |  |  |  |
| Cough |  |  |  |  |  |
| Vomit |  |  |  |  |  |
| Fever |  |  |  |  |  |
| Ear pain |  |  |  |  |  |

| 1. How often would you give your child antibiotics without the pediatricians’ advice, for the following reasons? | Always (95-100%) | Most of the times  (70-95%) | Often (30-70%) | Sometimes  (5-30%) | Never (0-5%) |
| --- | --- | --- | --- | --- | --- |
| Ι) Because you did not have enough spare time to visit a pediatrician, or because you did not have enough money to pay the visit. |  |  |  |  |  |
| ΙI) Because you thought that your child’s condition was not serious enough. |  |  |  |  |  |
| III) Because your pediatrician had prescribed the same antibiotic in the past, for the same symptoms. |  |  |  |  |  |
| IV) Because a pharmacist recommended the antibiotic. |  |  |  |  |  |
| V) Because a friend/ family relative recommended the antibiotic. |  |  |  |  |  |

|  | Strongly agree | Agree | Uncertain | Disagree | Strongly disagree |
| --- | --- | --- | --- | --- | --- |
| 1. Do you believe that antibiotics are used too much and unecessarily? |  |  |  |  |  |
| 1. Would you change your pediatrician because of not prescribing as many antibiotics as you think he/she should? |  |  |  |  |  |
| 1. Would you change your pediatrician because whenever you visit him/her, he/she keeps prescribing antibiotics? |  |  |  |  |  |
| 1. Would you reuse any leftover antibiotics whenever your child presents with similar symptoms of a URTI (i.e sore throat, flue, cold)? |  |  |  |  |  |
| 1. Do you think that parents and pediatricians should be informed about judicious antibiotic use? |  |  |  |  |  |
| 1. Do you think that most of the Upper Respiratory Tract Infections (i.e flue, cold, ear infection) resolve without antibiotic administration because they are self - limited? |  |  |  |  |  |
| 1. Would you request an antibiotic presciption if your child suffers from frequent Upper Respiratory Tract Infections? |  |  |  |  |  |
| 1. Would you visit a pediatrician if your child suffers just from runny nose? |  |  |  |  |  |
| 1. Do you think that you are worried about your child's health more than other parents do ? |  |  |  |  |  |
| 1. Would you visit a pediatrician in order to prevent any potential complications of your child's Upper Respiratory Tract Infection? |  |  |  |  |  |

**Section C**

|  | Very much | Plenty | Not much | A little | None |
| --- | --- | --- | --- | --- | --- |
| 1. Do you believe that you are well informed about judicious antibiotic use? |  |  |  |  |  |
| 1. How many antibiotics do you think your child receives in comparison to other children? |  |  |  |  |  |
| 1. How much do you consider the possible antibiotic adverse reactions when using them? |  |  |  |  |  |
| 1. Do you agree that you will be dissatisfied if your paediatrician does not prescribe antibiotics for Upper Respiratory Tract Infections (i.e. cold, ear infection, cough)? |  |  |  |  |  |

|  | Always (95-100%) | Most of the times  (70-95%) | Often (30-70%) | Sometimes  (5-30%) | Never (0-5%) |
| --- | --- | --- | --- | --- | --- |
| 1. Ηow often do you ask your pediatrician whether or not the prescription of antibiotics is necessary ? |  |  |  |  |  |
| 1. How often do you congratulate on your pediatrician' s not prescribing antibiotics? |  |  |  |  |  |
| 1. How often does your pediatrician recommend antibiotics on the phone? |  |  |  |  |  |
| 1. How often do you ask directly your paediatrician to prescribe antibiotics? |  |  |  |  |  |
| 1. How often do you completely follow all the pediatrician’s instructions and advice? |  |  |  |  |  |
| 1. How often do you insist on your pediatrician' s prescribing antibiotics as a precaution even if any diagnosis is not confirmed? |  |  |  |  |  |
| 1. How often does your pediatrician inform you about your child’s disease and notifies you whether it is necessary or not to receive antibiotics? |  |  |  |  |  |
| 1. How often do you think that your pediatrician prescribes antibiotic only because you asked him to? |  |  |  |  |  |
